# Tenesmus: An Unusual Presentation of Delayed Prostate Adenocarcinoma Recurrence

**DOI:** 10.7759/cureus.16609

**Published:** 2021-07-25

**Authors:** Mohammad Abdulelah, Nada Hajjaj, Mohammed A Abu-Rumaileh, David Clanon, Husam Bader

**Affiliations:** 1 Internal Medicine, University of Jordan School of Medicine, Amman, JOR; 2 Endocrinology, University of Jordan School of Medicine, Amman, JOR; 3 Internal Medicine, University of New Mexico, Albuquerque, USA; 4 Internal Medicine, Presbyterian Medical Center, Albuquerque, USA

**Keywords:** prostate cancer recurrence, tenesmus due to prostate cancer, psa elevation post radical prostatectomy, prostate cancer, prostate-specific antigen (psa), tenesmus

## Abstract

We describe a case of prostate cancer recurrence 25 years after radical prostatectomy. Our patient is a 77-year-old male with past medical history pertinent for obesity and coronary artery disease. The patient’s initial presentation in 1994 was for persistent lower urinary tract symptoms. He was subsequently diagnosed with high-grade prostate adenocarcinoma and underwent radical prostatectomy. The patient was followed up postoperatively for 16 years and deemed to be in clinical and biochemical remission with undetectable prostate-specific antigen (PSA).

Twenty-five years post-operatively, the patient was evaluated with an investigatory colonoscopy for tenesmus, constipation, and change in stool caliber. Colonoscopy revealed significant anal canal stenosis. Biopsy of the lesion showed prostate adenocarcinoma recurrence.

Prostate cancer recurrence presenting with only gastrointestinal symptoms is highly unusual, especially in a patient who never received radiotherapy and had been in remission for 25 years.

## Introduction

Prostate cancer, which is the most common malignancy in men, tends to remain unnoticed until urological symptoms arise. Symptoms commonly include hesitancy, urgency, incontinence, poor urinary stream, post-void dribbling, and nocturia. Current guidelines suggest management according to the severity and aggressiveness of the tumor. Therapeutic modalities include radical prostatectomy, radiotherapy, androgen deprivation therapy as well as cryosurgery [1]. 

Disease recurrence has become a more common entity nowadays, likely due to the accelerated trajectory in medical advances leading to improved overall quality of care and increased average life expectancy. This evolving matter requires physicians to be more highly attentive to patients with a history of prostate cancer despite how prolonged the disease-free period is or the initial modality of treatment and its expected adverse effects, as disease recurrence itself is an established cause of severe morbidity, mortality, and emotional debility. Fear of disease recurrence can be a taxing and stressful part of cancer survivorship, this dreaded possibility leaves physicians with the responsibility of appropriately and diligently evaluating for cancer recurrence. Multiple models are being developed and tested to predict disease recurrence and patient survival. Nevertheless, a clinician’s expertise along with specific patient risk factors remain the major determining factors in this field.

## Case presentation

**Figure 1:**
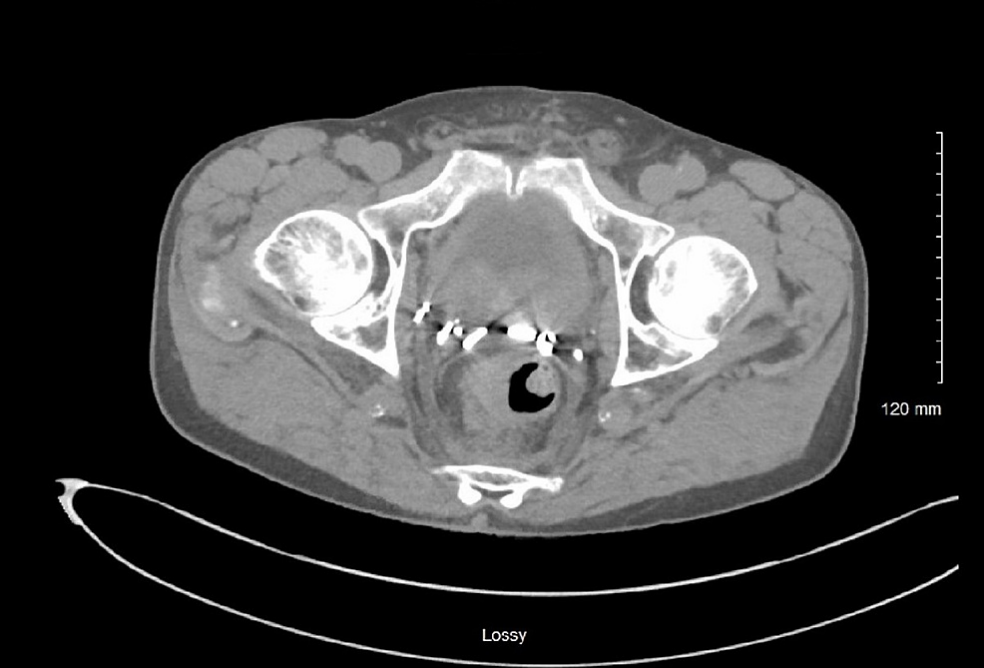
Pelvic CT scan revealing rectal wall thickening.

**Figure 2:**
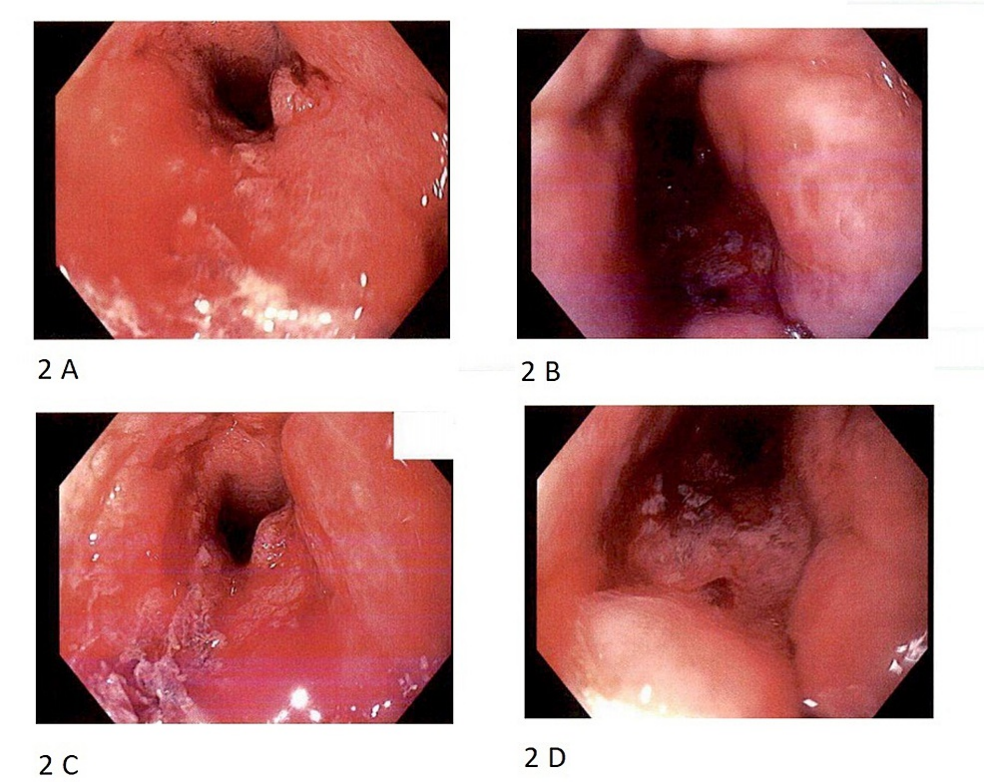
Colonoscopy images of an erythematous and stenotic anal canal. The image demonstrates the colonoscopy findings of the patient. (A and B) The stenotic regions; (C and D) the erythematic and friable mucosal lining.

A 77-year-old male with a relevant past medical history of high-grade prostate cancer managed by radical prostatectomy in 1994 was referred to our tertiary medical center for evaluation of GI symptoms. The patient was evaluated by our gastrointestinal team for worsening constipation, incomplete sense of defecation, and change in the caliber of stool over a period of nine months. Computed tomography (CT) scan of the abdomen and pelvis noted significant anal and rectal wall thickening (Figure 1). Subsequent diagnostic colonoscopy showed circumferential malignant appearing anal stenosis, with friable, erythematous mucosa (Figure 2). Biopsies of the anal lesion were positive for invasive poorly differentiated adenocarcinoma. MLH-1/PMS-2/MSH-2/MSH-6 were positive (proteins expressed), and thus, ruling out genetic predisposition syndromes.

The patient was referred to oncology. Records of his medical care between 1994 and 2010 were reviewed and included a prostate-specific antigen (PSA) of 9 ng/ml at the time of the first diagnosis in 1994, treatment with a retropubic nerve-sparing radical prostatectomy, and prostatic lymph nodes obtained intraoperatively at the time were negative for malignancy. The patient did not receive any radiotherapy or androgen deprivation therapy. Follow-up PSA over the following 16 years did not exceed 0.06 ng/ml.

At the time of referral to oncology, PSA was 155 ng/ml. CT scan of the chest, abdomen, and pelvis was ordered as part of workup for recurrent prostate cancer. The CT showed moderate rectal wall thickening, few new sub-centimeter sclerotic foci in the bilateral ribs and right scapula concerning osseous metastatic disease, and mildly enlarged abdominal and pelvic lymph nodes. Additional urologic findings included bladder wall thickening with a new moderate left hydronephrosis and hydroureter and a new mild right hydroureter. The cause of these findings was suggested to be either result of local recurrence or sequelae of chronic outlet obstruction and postprocedural changes.

A care plan for the patient was established by a multidisciplinary team. Treatment consisted of triple therapy including bicalutamide, enzalutamide, leuprorelin as well as multiple laxatives for constipation and tenesmus. The patient received radiation therapy for further control of symptoms. The patient tolerated therapy well with satisfactory outcomes and intermittent improvement of tenesmus.

## Discussion

This case report discusses a case of prostate cancer recurrence 25 years after radical prostatectomy. It illustrates the importance of continual monitoring and maintaining a high clinical suspicion of recurrence if any unusual or subtle symptoms arise, irrespective of the disease-free duration. Biochemical recurrence could be as prevalent as 33% in those who undergo radical prostatectomy which might make a case for more strict adjuvant hormonal therapy [2,3]. Common traits linking disease recurrence have been studied extensively in the past. Researchers from the caPURSE study found that morbidly obese patients (BMI 35 kg/m^2^ or more) were 1.69 times more likely to have recurrence relative to men of normal weight, 95% confidence interval (CI) 1.01 to 2.84. Ethnicity was not linked to recurrence [4]. Furthermore, a different study found that smoking one year after prostatectomy was associated with a more than two-fold increased risk of recurrence [5].

Various signs and symptoms have been known to raise suspicion about possible recurrence of prostate cancer after surgery, however, gastrointestinal manifestations are uncommon [6]. Our patient primarily complained of tenesmus and constipation. Those symptoms could have been easily mistaken for local diseases of the GI tract and prostate cancer recurrence could have been easily overlooked in light of the prolonged disease-free period. In addition, despite the fact that our patient did not receive radiotherapy, colonoscopic features revealed stenosis of the anal canal which is one of the well-established long-term severe complications in patients who undergo radiotherapy [7].

This is the first case to the authors’ knowledge that combines both an abnormal presentation as well as a very elongated time to recurrence. Therefore, we encourage clinicians to expand their differential diagnoses and consider recurrence when evaluating patients with a previous history of treated prostate cancer, as the earlier diagnosis can expedite the control of disease and improve quality of life. Additionally, marked advances in imaging of both local and distant tumor recurrences can now allow a specific selection of local and systemic treatment options tailored to patients and their disease with less associated morbidity [8].

## Conclusions

The authors believe in the importance of patient education regarding the significance of regular follow-up, and establishing a common understanding that recurrence should not be overlooked. We also encourage a lower threshold in considering recurrence regardless of the duration of the disease-free period in patients who are at higher risk with suggestive symptomology. In addition, regular monitoring and testing should be executed in order to decrease possible future morbidity and mortality as cancer recurrence affects one-third of those who undergo treatment for prostate cancer. The feasibility of more advanced screening methods, perhaps regular testing with transrectal ultrasound, should be considered for certain patients with the established risk factors for recurrence. Furthermore, uncommon presentations of recurrence could include anal canal stenosis presenting as tenesmus and decreased stool output even in the absence of previous exposure to radiotherapy.

Further studies are required to evaluate the benefit of neoadjuvant and adjuvant therapies, in those with established risk factors for recurrence, in addition to standard of care for possible improvement in patient care, disease-free outcomes, and a decrease in recurrence rate.
